# Determination of Solubility Parameters of Ibuprofen and Ibuprofen Lysinate

**DOI:** 10.3390/molecules201219777

**Published:** 2015-12-03

**Authors:** Teja Kitak, Aleksandra Dumičić, Odon Planinšek, Rok Šibanc, Stanko Srčič

**Affiliations:** 1 Faculty of Pharmacy, University of Ljubljana, Aškerčeva cesta 7, 1000 Ljubljana, Slovenia; teja.kitak@gmail.com (T.K.); odon.planinsek@ffa.uni-lj.si (O.P.); rok.sibanc@ffa.uni-lj.si (R.Š.); 2 Zenitva k.s., U Kabelovny 130, 102 37 Prague 10, Czech Republic; aleksandra.dumicic@zentiva.cz

**Keywords:** Hansen’s solubility parameters, extended Hansen’s approach, inverse gas chromatography, ibuprofen, ibuprofen lysinate

## Abstract

In recent years there has been a growing interest in formulating solid dispersions, which purposes mainly include solubility enhancement, sustained drug release and taste masking. The most notable problem by these dispersions is drug-carrier (in)solubility. Here we focus on solubility parameters as a tool for predicting the solubility of a drug in certain carriers. Solubility parameters were determined in two different ways: solely by using calculation methods, and by experimental approaches. Six different calculation methods were applied in order to calculate the solubility parameters of the drug ibuprofen and several excipients. However, we were not able to do so in the case of ibuprofen lysinate, as calculation models for salts are still not defined. Therefore, the extended Hansen’s approach and inverse gas chromatography (IGC) were used for evaluating of solubility parameters for ibuprofen lysinate. The obtained values of the total solubility parameter did not differ much between the two methods: by the extended Hansen’s approach it was δt = 31.15 MPa^0.5^ and with IGC it was δ_t_ = 35.17 MPa^0.5^. However, the values of partial solubility parameters, *i.e.*, δ_d_, δ_p_ and δ_h_, did differ from each other, what might be due to the complex behaviour of a salt in the presence of various solvents.

## 1. Introduction

One of the main challenges in the fields of formulation development is the formulation of new and outstanding products that will meet the required regulatory and market criteria. The limits that often prevent the success of such goals may occur at various steps of product development. In this work we focused on how to overcome the limits which often occur during pre-formulation studies and are closely related to the solubility of a drug with excipients. One way of determining whether the drug is miscible with a selected excipient is the use of a parameter, called solubility parameter. Initially, the idea of solubility parameter was introduced by Hildebrand and Scott and was based on an estimation of the solubility behavior of substances [1,2]. Hildebrand defined the solubility parameter, δ_t_, as square root of the cohesive energy density (CED) of a substance. CED is an energy required to separate the atoms or molecules from each other and it’s a direct criterion of attractiveness [3]:

(1) $$\delta_{t}={\left(\text{CED}\right)}^{0.5}={\left(\frac{{\Delta H}_{v}-\text{RT}}{V_{m}}\right)}^{0.5}$$

where ΔH_v_ is the heat of vaporization, R is the gas constant, T is the temperature and V_m_ is the molar volume.

This concept was originally developed for simple liquid mixtures and can be used in various practical applications for this reason. However, it can’t meet the needs of more complex systems, as are typical pharmaceutical substances [4]. A number of approximations and extensions of Hildebrand’s concept have been made due to better understanding of the complex interactions between substances. The most established is the so-called Hansen approach [5,6,7]. The basis of his theory lies within dividing total cohesive energy, E_coh_, on components arising from dispersion forces, E_d_, permanent dipole-dipole interactions, E_p_, and hydrogen bonds, E_h_ [8]:

(2) $$E_{\text{coh}}=E_{d}+E_{p}+E_{h}$$

If the equation above is divided by molar volume, V_m_, and squared, it gives us a new three dimensional approach in which Hildebrand’s total solubility parameter squared is equal to the sum of the squares of Hansen’s components [8]:

(3) $$\delta_{t}^{2}=\delta_{d}^{2}+\delta_{p}^{2}+\delta_{h}^{2}$$

where δ_d_, δ_p_ and δ_h_ are Hansen’s solubility parameters which represent the three main types of interactions in organic matter.

The most commonly studied interactions in the pharmaceutical industry are interactions related to solubility, where the greatest interest lies in solubility interactions between solvent and polymer, solvent and drug and also polymer and drug. For this purpose, a number of approaches based on solubility parameter theory or “like dissolves like”, have been developed.

Van Krevelen and Hoftyzer used the parameter $\Delta \bar{\delta }$ to describe mixing between two substances [3]:

(4) $$\Delta \bar{\delta }={\left[{\left(\delta_{d2}-\delta_{d1}\right)}^{2}+{\left(\delta_{p2}-\delta_{p1}\right)}^{2}+{\left(\delta_{h2}-\delta_{h1}\right)}^{2}\right]}^{0.5}$$

The threshold value at which there is a high probability of miscibility between two substances, was determined at $\Delta \bar{\delta }\leq 5\;{\text{MPa}}^{0.5}$ [3,9]. Primarily, miscibility was only considered for a solution with a constant concentration of polymer as a solute [3,9,10]. However, recent studies have brought the usefulness of the parameter $\Delta \bar{\delta }$ to completely different level, by determining the degree of mixing between active substance and various excipients [11].

On the other hand, Bagley [12] presented the theory of thermodynamic similarity between δ_d_ and δ_p_, and introduced new, combined solubility parameter, δ_v_:

(5) $$\delta_{v}={\left(\delta_{d}^{2}+\delta_{p}^{2}\right)}^{0.5}$$

By using this approach one can project the three-dimensional solubility parameter space into a two-dimensional plot. Plot δ_v_ as a function of δ_h_ is called Bagley’s plot. The distance, R_a(v)_, in the Bagley’s plot, can be expressed by the following equation:

(6) $$R_{a\left(v\right)}={\left[4{\left(\delta_{v2}-\delta_{v1}\right)}^{2}+{\left(\delta_{h2}-\delta_{h1}\right)}^{2}\right]}^{0.5}$$

and represents the level of solubility/miscibility between substances. Bagley plots have proved especially useful in the case of determining solubility between polymers and solvents [3], but other studies [11,13] also prove its usefulness in determining miscibility between an active substance and an excipient or polymer. In the case of miscibility, the value of the parameter R_a(v)_ should be in the range of R_a(v)_ ≤ 5.6 MPa^0.5^.

It seems like the most often used approach in determining miscibility between two components, is Greenhalgh’s approach. In order to define miscibility, Greenhalgh *et al.* [14] used the difference in values of total solubility parameters, δ_t_:

(7) $${\Delta \delta }_{t}=\left|\delta_{t2}-\delta_{t1}\right|$$

The results of many combinations of active substances and excipients gave a clear trend of solubility at Δδ_t_ < 7 MPa^0.5^. They were also able to estimate the difference of total solubility parameters at which the mixing is not likely to occur, Δδ_t_ > 10 MPa^0.5^ [14]. Numerous studies have confirmed the validity of Greenhalgh’s approach [11,13,15,16,17], however, on the other hand some studies [18,19] have pointed out the deficiency of comparisons between total solubility parameters.

The total solubility parameter, δ_t_, for solvents, polymers and active substances, can be determined by many different ways, using different methods, all being applied in the existing literature. In order to obtain greater insight on interactions between substances, it is crucial to divide the total solubility parameter into the individual contributions (δ_d_, δ_p_ and δ_h_), but there are only few methods for determination of all three partial solubility parameters. These correlations are still under research. In particular, the biggest limitation is the determination of partial solubility parameters of a drug, since most methods, used in the case of solvents and polymers, cannot be applied to drug molecules [20]. In this work we focused on determination of solubility parameters, using two different approaches: solely by calculation and an experimental approach.

### 1.1. Calculation Methods

There are number of approaches for the calculation of solubility parameters of a substance by using group contribution methods. All of them share the same theory that the total cohesive energy density of a molecule is an additive property and it is the sum of contributions from individual functional groups within the molecule [21]. The main advantage is the fast and easy way of determination of dispersive, polar and hydrogen bonding solubility parameters by having an adequate knowledge of molecular structure. Furthermore, numerous references demonstrate the credibility of solubility parameter calculations for various excipients, with a predominance of polymers, and several drugs [3,8,11,13,14,17,21,22]. Group contribution methods may be accurate and time-saving methods, however, their usage is limited. One main disadvantage is the absence of the data needed for the calculation of solubility parameters of active salts [23].

### 1.2. Experimental Approach

#### 1.2.1. Extended Hansen’s Approach (EHA)

The extended Hansen approach is a method that uses experimental data in a regression model in order to obtain values of solubility parameters of selected substances. Bustamante *et al.*, complemented the original extended Hansen approach [5,24] and used a regression model that links the solubility parameters of solvents and ln_χ2_, with the solubility parameters of a drug:

(8) $$ln\;\chi_{2}=C_{0}+C_{1}\delta_{1d}^{2}+C_{2}\delta_{1d}+C_{3}\delta_{1p}^{2}+C_{4}\delta_{1p}+C_{5}\delta_{1h}^{2}+C_{6}\delta_{1h}$$

where the numbers 1 and 2 refer to the solvent and solute, respectively, C_0–6_ are the coefficients of the regression analysis and ln_χ2_ is a mole fraction solubility of a drug in a given solvent. Partial solubility parameters are then calculated using the constants of the regression analysis, C_0–6_:

(9) $$\delta_{2d}=-\left(\frac{C_{2}}{2C_{1}}\right)$$

(10) $$\delta_{2p}=-\left(\frac{C_{4}}{2C_{3}}\right)$$

(11) $$\delta_{2h}=-\left(\frac{C_{6}}{2C_{5}}\right)$$

#### 1.2.2. Inverse Gas Chromatography (IGC)

With the development of more and more complex technological processes, which enable more sophisticated pharmaceutical dosage forms, a tendency towards alternative methods of evaluation of physiochemical properties of solid pharmaceutical substances has arisen. Thus, in 1967 a modified form of gas chromatography, called inverse gas chromatography (IGC) was developed [25]. Unlike its conventional form, with the IGC method a solid sample is placed in a chromatography column, which represents the stationary phase. The stationary phase is washed with the mobile phase, which is represented as an inert gas, such as nitrogen or helium. Into the flow of inert gas are injected vapor phases of different organic solvents with known characteristics. The extent of interactions between the vapor of a selected solvent and solid sample is reflected in a specific retention volume, V_g_. Once the value of V_g_ is obtained, chromatographic data can be converted into thermodynamic parameters using Equation (12) [26]:

(12) $$\chi_{1,2}^{\infty }=\text{ln}\left(\frac{273.15R}{p_{1}^{0}V_{g}M_{r,1}}\right)-\frac{p_{1}^{0}}{\text{RT}}\left(B_{11}-V_{1}\right)+\text{ln}\left(\frac{\rho_{1}}{\rho_{2}}\right)-\left(1-\frac{V_{1}}{V_{2}}\right)$$

where the numbers 1 and 2 refer to the solvent and solute, respectively, and M_r,1_, $p_{1}^{0}$, B_11_, V_g_, V_1_, V_2_, ρ_1_, ρ_2_, represent the molecular mass, saturated vapor pressure, second virial coefficient, specific retention volume, molar volume and density of the solvent and solute, respectively.

There is a linear correlation between $\chi_{\left(1,2\right)i}^{\infty }$ and δ_1i_:

(13) $$\frac{\delta_{1i}^{2}}{\text{RT}}-\frac{\chi_{\left(1,2\right)i}^{\infty }}{V_{1}}=\frac{2\delta_{2}}{\text{RT}}\delta_{1i}-\left(\frac{\delta_{2}^{2}}{\text{RT}}+\frac{\chi_{s}^{\infty }}{V_{1}}\right)$$

where $\frac{2\delta_{2}}{RT}$ represents the inclination of the straight line and is proportional with the solubility parameter of the substance, δ_2_. Based on the given equation, δ_2_ can also be calculated from the intersection with the abscissa $\left(\frac{\delta_{2}^{2}}{RT}+\frac{\chi_{s}^{\infty }}{V_{1}}\right)$.

Voelkel and Janas [27] upgraded the already known Price method [28] for the calculation of Hansen solubility parameters, and thereby contributed to the usefulness of IGC in determination of partial solubility parameters for solid substances [29,30,31,32]. In order to obtain values of partial solubility parameters, tested organic solvents, with known properties, must be classified regarding to their ability for intermolecular interactions. Solvents are divided into three groups, representing dispersion, polar and hydrogen bonding interactions. By using Equation (13) for each group of solvents, one can obtain three straight lines in graphical representation. Partial solubility parameters can then be calculated from the slope of the straight line, using [27]:

(14) $$\delta_{d}=\frac{m_{n-\text{alkanes}}RT}{2}$$

(15) $$\delta_{p}=\frac{\left(m_{1}-m_{n-\text{alkanes}}\right)RT}{2}$$

(16) $$\delta_{h}=\frac{\left(m_{2}-m_{n-\text{alkanes}}\right)RT}{2}$$

where *m*_*n*-alkanes_ represents the value of the slope for the group of *n*-alkanes, m_1_ represents the value of the slope for the group of polar solvents (aromatic hydrocarbons, ketones, 1-nitropropane, acetonitrile, 1,2-dichloroethane) and m_2_ represents the value of the slope for the group of solvents with the ability to form hydrogen bonds (alcohols, pyridine, 1,2-dioxane).

The crucial step in partial solubility determination, by using IGC, is selection of the solvents. In the case of a group of solvents that can form only dispersive interactions, the choice is rather simple. The chosen *n*-alkanes only show this type of interaction. However, this is not the case with the selection of the solvents in the other two groups. The criteria are not always precise, as in general, organic solvents can interact through both polar interactions and hydrogen bonding. One of the most commonly used criteria is the ratio between δh and δp. The higher the ratio, δp/δh, the more appropriate is the solvent to be classified in the polar solvents group. The same applies to the ratio δh/δp and the classification of solvents in the group which is able of forming hydrogen bonds [31].

The following work considers a classical and universally accepted concept of physical chemistry, the solubility parameter. The basis of solubility parameter theory lies within similarity or “like dissolves like”. However, the reader should be aware that the principle of similarity is often not sufficient enough to evaluate the solubility of a solute in a solvent. Other principles, such as complementarity, should also be taken into consideration. Similar observations have been previously stated on the interactions between the surfaces of liquids, solids-liquids and solids-solids as well.

Fowkes [33] noted that surface interactions could only occur between surfaces of similar type; for example no interaction due to the permanent dipoles can take place across an interface between polar and non-polar materials. This is the similarity principle. However, this concept failed to predict interfacial interactions between certain liquids. Van Oss *et al.* [34] described difficulties in considering polar interactions as all being of a similar type, and he pointed out that there are materials which could be described as polar, which, however, were dipolar, hydrogen bonding, Lewis acids, or Lewis bases. Considering the polar materials as either electron donor or electron acceptors explains why two polar materials can repel each other, when they are of a monopolar type. The same principle might be used in solubility observations as well.

There is therefore a continuing effort towards new definitions and understanding, which account for both similarity and complementarity components and thus can overcome some of the restrictions of the original definition. However, in this work we are still only using the principle of similarity approach and therefore the complementarity principle is not a part of further discussion.

The issue of further discussion is predicting the solubility of a given drug and its salt in polymers and non-aqueous solvents. By taking knowledge about the solubility of a drug into account, one can overcome limits, which arise during manufacturing process, as well as other stressful situations.

In this work, solubility parameters were chosen as potential parameters for predicting, estimating and evaluating the solubility of ibuprofen lysinate and the main goal was their determination.

## 2. Results

### 2.1. Calculation Methods

#### 2.1.1. Ibuprofen

Solubility parameters of ibuprofen were calculated, using six different group contribution methods. The results are summarized in Table 1.

**Table 1:** Values of solubility parameters for ibuprofen by using six different group contribution methods.

Method						
Hansen and Beerbower	Fedors	Hoftyzer and Van Krevelen	Hoy	Stefanis and Panayiotou	Just and Breitkreutz	Median δ_t_ (MPa^0.5^)
δ_t_ = 19.89	δ_t_ = 20.91	δ_t_ = 19.36	δ_t_ = 19.71	δ_t_ = 19.7	δ_t_ = 19.20	δ_t_ = 19.71
		δ_d_ = 17.85		δ_d_ = 17.56	δ_d_ = 16.59	
		δ_p_ = 2.22		δ_p_ = 3.22	δ_p_ = 4.27	
		δ_h_ = 7.15		δ_h_ = 8.31	δ_h_ = 8.67	

Total solubility parameter values were consistent throughout the calculations, regardless to the method chosen. The lowest value of the total solubility parameter, δ_t_ = 19.20 MPa^0.5^, was obtained by using the group contribution method of Just and Breitkreutz. The highest value, δ_t_ = 20.91 MPa^0.5^, was obtained when using group contribution method of Fedors. The partial solubility parameters values also did not differ much between methods. The largest value in the case of the dispersion solubility parameter was obtained by using the group contribution method of Hoftyzer and Van Krevelen and the smallest value using the method of Just and Breitkreutz, where the values were δ_d_ = 17.85 MPa^0.5^ and δ_d_ = 16.59 MPa^0.5^, respectively. The opposite trend had been noticed in the case of polar and hydrogen bonding solubility parameters, where the lowest values were obtained by using the group contribution method of Hoftyzer and Van Krevelen, δ_p_ = 2.22 MPa^0.5^ in δ_h_ = 7.15 MPa^0.5^, and the highest values using the method of Just and Breitkreutz, δ_p_ = 4.27 MPa^0.5^ in δ_h_ = 8.67 MPa^0.5^. In the following, the median of all six group contribution methods was considered as the value of the total solubility parameter of ibuprofen.

#### 2.1.2. Polymers and Excipients

Solubility parameters of some frequently used polymers and excipients were calculated using the same group contribution methods as in the case of ibuprofen. The results are summarized in Table 2.

**Table 2:** Values of solubility parameters for polymers and excipients by using six different group contribution methods.

Excipient	Method						
	Hansen and Beerbower	Fedors	Hoftyzer and Van Krevelen	Hoy	Stefanis and Panayiotou	Just and Breitkreutz	Median δ_t_ (MPa^0.5^)
Copovidone	δ_t_ = 19.01	δ_t_ = 22.89	δ_t_ = 24.37	δ_t_ = 20.25	δ_t_ = 22.39	δ_t_ = 21.24	δ_t_ = 21.82
			δ_d_ = 19.23		δ_d_ = 18.18	δ_d_ = 15.45	
			δ_p_ = 11.15		δ_p_ = 10.15	δ_p_ = 8.61	
			δ_h_ = 9.67		δ_h_ = 7.82	δ_h_ = 11.42	
Povidone K30	δ_t_ = 18.96	δ_t_ = 23.75	δ_t_ = 26.28	δ_t_ = 20.05	δ_t_ = 23.60	δ_t_ = 21.75	δ_t_ = 22.68
			δ_d_ = 20.44		δ_d_ = 19.05	δ_d_ = 16.67	
			δ_p_ = 13.67		δ_p_ = 12.09	δ_p_ = 9.85	
			δ_h_ = 9.28		δ_h_ = 6.86	δ_h_ = 9.9	
PEG 6000	δ_t_ = 18.38	δ_t_ = 19.17	δ_t_ = 22.86	δ_t_ = 21.44	δ_t_ = 20.45	δ_t_ = 17.76	δ_t_ = 19.81
			δ_d_ = 17.78		δ_d_ = 17.33	δ_d_ = 13.6	
			δ_p_ = 11.1		δ_p_ = 7.93	δ_p_ = 11.09	
			δ_h_ = 9.13		δ_h_ = 7.42	δ_h_ = 2.75	
Eudragit^®^ E PO	δ_t_ = 17.34	δ_t_ = 19.59	δ_t_ = 19.70	δ_t_ = 18.28	δ_t_ = 23.51	δ_t_ = 16.04	δ_t_ = 19.00
			δ_d_ = 17.35		δ_d_ = 16.90	δ_d_ = 12.19	
			δ_p_ = 3.06		δ_p_ = 15.45	δ_p_ = 1.89	
			δ_h_ = 8.81		δ_h_ = 5.31	δ_h_ = 10.26	
Eudragit^®^ S100	δ_t_ = 20.02	δ_t_ = 21.86	δ_t_ = 21.1	δ_t_ = 19.29	δ_t_ = 22.37	δ_t_ = 18.63	δ_t_ = 20.56
			δ_d_ = 18.01		δ_d_ = 16.31	δ_d_ = 12.18	
			δ_p_ = 3.64		δ_p_ = 12.3	δ_p_ = 4.54	
			δ_h_ = 10.38		δ_h_ = 9.11	δ_h_ = 13.34	
Poloxamer 188	δ_t_ = 18.30	δ_t_ = 18.81	δ_t_ = 22.10	δ_t_ = 20.9	δ_t_ = 20.33	δ_t_ = 17.31	δ_t_ = 19.57
			δ_d_ = 17.49		δ_d_ = 17.30	δ_d_ = 13.66	
			δ_p_ = 10.23		δ_p_ = 7.72	δ_p_ = 10.24	
			δ_h_ = 8.74		δ_h_ = 7.37	δ_h_ = 2.64	
Poloxamer 407	δ_t_ = 18.26	δ_t_ = 18.64	δ_t_ = 21.75	δ_t_ = 20.66	δ_t_ = 20.28	δ_t_ = 17.12	δ_t_ = 19.46
			δ_d_ = 17.36		δ_d_ = 17.29	δ_d_ = 13.68	
			δ_p_ = 9.85		δ_p_ = 7.63	δ_p_ = 9.89	
			δ_h_ = 8.57		δ_h_ = 7.35	δ_h_ = 2.58	
Methylcellulose (DS = 1)	δ_t_ = 27.05	δ_t_ = 29.78	δ_t_ = 32.29	δ_t_ = 27.23	δ_t_ = 24.82	δ_t_ = 27.45	δ_t_ = 27.34
			δ_d_ = 20.73		δ_d_ = 18.38	δ_d_ = 17.91	
			δ_p_ = 10.26		δ_p_ = 7.15	δ_p_ = 17.74	
			δ_h_ = 22.54		δ_h_ = 14.82	δ_h_ = 10.66	
Ethylcellulose (DS = 3)	δ_t_ = 15.86	δ_t_ = 18.79	δ_t_ = 20.18	δ_t_ = 20.1	δ_t_ = 19.58	δ_t_ = 21.42	δ_t_ = 19.84
			δ_d_ = 17.59		δ_d_ = 17.79	δ_d_ = 16.09	
			δ_p_ = 4.59		δ_p_ = 6.44	δ_p_ = 14.05	
			δ_h_ = 8.77		δ_h_ = 5.05	δ_h_ = 1.61	
Xylitol	δ_t_ = 36.86	δ_t_ = 40.60	δ_t_ = 41.53	δ_t_ = 36.60	δ_t_ = 30.56	δ_t_ = 31.3	δ_t_ = 36.73
			δ_d_ = 20.75		δ_d_ = 18.62	δ_d_ = 16.07	
			δ_p_ = 12.68		δ_p_ = 11.87	δ_p_ = 21.15	
			δ_h_ = 33.67		δ_h_ = 21.13	δ_h_ = 16.54	
Sucrose	δ_t_ = 35.81	δ_t_ = 39.95	δ_t_ = 40.38	δ_t_ = 33.53	δ_t_ = 40.83	δ_t_ = 31.15	δ_t_ = 37.88
			δ_d_ = 21.76		δ_d_ = 21.35	δ_d_ = 18.55	
			δ_p_ = 9.87		δ_p_ = 18.44	δ_p_ = 19.63	
			δ_h_ = 32.55		δ_h_ = 29.51	δ_h_ = 15.53	
Lactose	δ_t_ = 34.07	δ_t_ = 38.86	δ_t_ = 40.46	δ_t_ = 31.48	δ_t_ = 37.45	δ_t_ = 38.94	δ_t_ = 38.16
			δ_d_ = 22.62		δ_d_ = 21.78	δ_d_ = 20.07	
			δ_p_ = 9.63		δ_p_ = 19.71	δ_p_ = 27.90	
			δ_h_ = 32.14		δ_h_ = 23.14	δ_h_ = 18.30	

The values of the total solubility parameter for each polymer and excipient did not differ significantly between different methods. The highest value was obtained using the group contribution method of Hoftyzer and Van Krevelen, in eight out of twelve cases. The lowest value showed up most often (in six out of twelve cases) when the method of Just and Breitkreutz was used.

In Table 2 it is clearly seen that the use of different methods gives different partial solubility parameter trends. In the case of dispersion solubility parameter the trend is similar to that of total solubility parameter. It doesn’t vary significantly between methods. The highest values are obtained using the group contribution method by Hoftzyer and Van Krevelen, and the lowest values are obtained using the method by Just and Breitkreutz, in all cases. The highest values of the polar solubility parameter were obtained using the method by Just and Breitkreutz in most cases and the lowest values using the method by Hoftyzer and Van Krevelen. The opposite trend was noticed in the case of calculating the values of the hydrogen bonding solubility parameter. The main reason for the deviation between the results is the fact that different group contribution methods evaluate the same increments differently. For example, the increment –O– has a lower value for the polar solubility parameter contribution, Fpi, and a higher value for the hydrogen bonding solubility parameter contribution, Ehi, when comparing the group contribution method authored by Hoftyzer and Van Krevelen to the one by Just and Breitkreutz.

In what follows, the median of all six group contribution methods was considered as a value of the total solubility parameter of selected excipients and polymers. In the case of partial solubility parameters averaging was not taken into consideration, because of the significant deviation among methods. For this reason, further processing of partial solubility parameters considers the results from all calculations.

As mentioned in the introduction, values of the solubility parameters of ibuprofen lysinate cannot be determined using group contribution methods. Therefore, the use of experimental approaches was needed.

### 2.2. Experimental Approach

#### 2.2.1. Extended Hansen’s Approach (EHA)

The Extended Hansen approach was already applied to ibuprofen and its salt, sodium ibuprofenate, in order to obtain solubility parameter values [35]. However, it has not been used for the determination of solubility parameters of a complex salt, like ibuprofen lysinate. The aim of this work was therefore to obtain the solubility parameters of ibuprofen lysinate, by using EHA.

**Figure 1:**
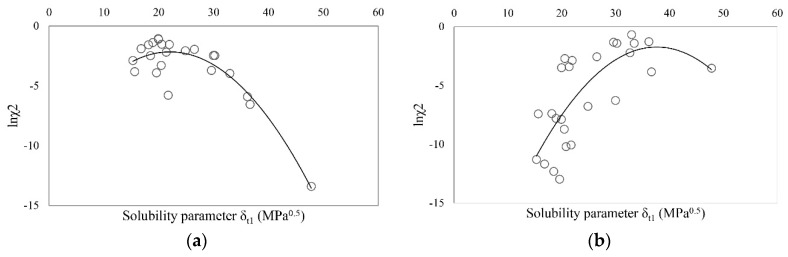

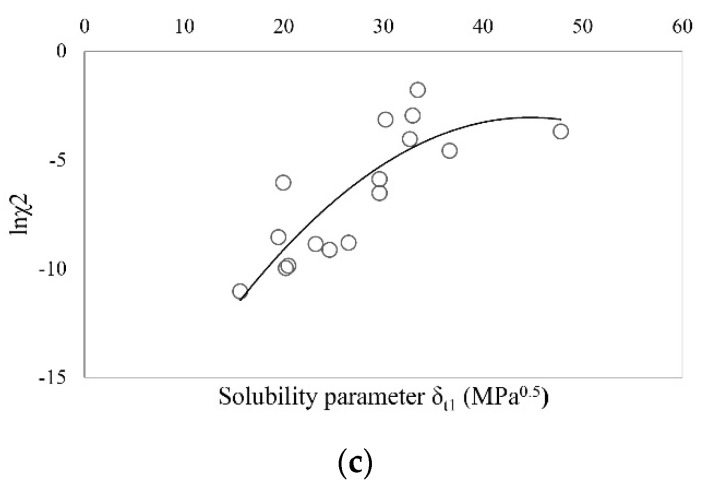
ln_χ2_ as a function of total solubility parameter for (**a**) ibuprofen; (**b**) sodium ibuprofen and (**c**) ibuprofen lysinate.

The solubility results, converted into mole fraction units, are listed in Table 3, together with the mole fraction solubility for ibuprofen and sodium ibuprofen, taken from [35]. The difference in solubility between all processed drug substances can be seen in Figure 1. The graphs can serve us as criterion of polarity and also for qualitative estimation of maximum solubility of a substance in the tested solvents. Since “like dissolves like” is a basis of solubility parameter theory, one can roughly estimate solubility parameters of given substance by finding the area of maximal solubility (Figure 1).

For further evaluation of solubility parameters, the experimental results, ln_χ2_, from the aforementioned procedure were also used in the regression model. By using simple linear regression for the solubility of a given drug, in all solvents, it was not possible to obtain an appropriate calculation procedure for determination of solubility parameters. Also, several types of robust regression, carried out in the statistical program R, gave us results that differ significantly. For this reason, an entirely different approach for processing the experimental data was chosen.

**Table 3:** Experimental determination of solubility, ln_χ2_, for ibuprofen and sodium ibuprofen (data taken from [35]) and ibuprofen lysinate.

Solvent	δ_t_ (MPa^0.5^)	Ibuprofen	Sodium Ibuprofen	Ibuprofen Lysinate
		lnχ_2_		
Ethanol	26.52	−1.9503	−2.5573	−8.8013
Chloroform	18.95	−1.3712	−7.7910	/
Methanol	29.61	−3.7017	−1.3116	−5.8776
Benzene	18.51	−2.4684	−12.3017	/
Dioxane	20.47	−3.2918	−8.7174	−9.8535
Acetic acid	21.37	−2.1746	−3.3999	−3.0117
1-Pentanol	21.93	−1.5436	−2.8702	/
1-Butanol	23.20	/	/	−8.8584
1-Propanol	24.60	/	/	−9.1191
Cyclohexane	16.80	−1.8834	−11.6705	/
1,2-Propanediol	30.22	−2.4547	−1.4146	−3.1307
Formamide	36.65	−6.5485	−3.8359	−4.5675
Ethylene glycol	32.95	−3.9612	−0.6762	−2.9535
Glycerol	36.16	−5.8932	−1.2651	/
Ethyl acetate	18.15	−1.0942	−7.3852	−9.6817
Propionic acid	19.95	−1.5611	−3.4886	/
1-Octanol	20.56	−1.5134	−2.7018	/
Heptane	15.30	−2.8827	−11.2964	−9.1165
Chlorobenzene	19.58	−3.9057	−12.9644	/
Diethyl ether	15.64	−3.8065	−7.4103	−11.039
Acetone	19.94	−1.0474	−7.8829	−6.0326
Acetophenone	21.72	−5.7807	−10.0583	/
*N,N*-Dimethylformamide	24.86	−2.058	−6.7721	/
Dichloromethane	20.20	/	/	−9.9672
Water	47.81	−13.398	−3.5326	−3.6882
*N*-Methylformamide	29.98	/	−6.2656	/
1,4-Butanediol	33.44	/	−1.4053	/
1,3-Propanediol	32.65	/	−2.2121	/
Ethylene dichloride	20.80	−2.4549	−10.1933	/
Tetrahydrofuran	23.20	/	/	8.5397

Solubility parameters of ibuprofen lysinate were calculated using linear regression, for all possible combinations of eight different solvents among all tested solvents, in Wolfram Mathematica. The mathematical procedure of linear regression and combination of eight different solvents did not always deliver reasonable results. Therefore, unreasonable results such as negative values of solubility parameters were defined as failed combinations or errors. In Table 4 data on the number of combinations processed for each individual drug and the number of failed combinations/errors, are shown. Once all of the values of solubility parameters for all of combinations were obtained, the median value for each solubility parameter of given drug was calculated. The results are summarized in Table 4.

**Table 4:** The values of solubility parameters for ibuprofen, sodium ibuprofen and ibuprofen lysinat obtained by using EHA.

Drug	Solubility Parameter (MPa^0.5^)				Analysis Parameters			
	δ_t_	δ_d_	δ_p_	δ_h_	No. of Solvents	No. of Combinations	No. of Errors	R^2^
Ibuprofen	20.56	16.60	6.91	9.97	23	490,314	109,416	0.9964
Sodium ibuprofenate	34.12	16.38	12.52	27.19	26	1,562,275	220,884	0.9964
Ibuprofen lysinate	31.15	16.97	22.75	12.83	16	12,870	1573	0.7594

**Figure 2:**
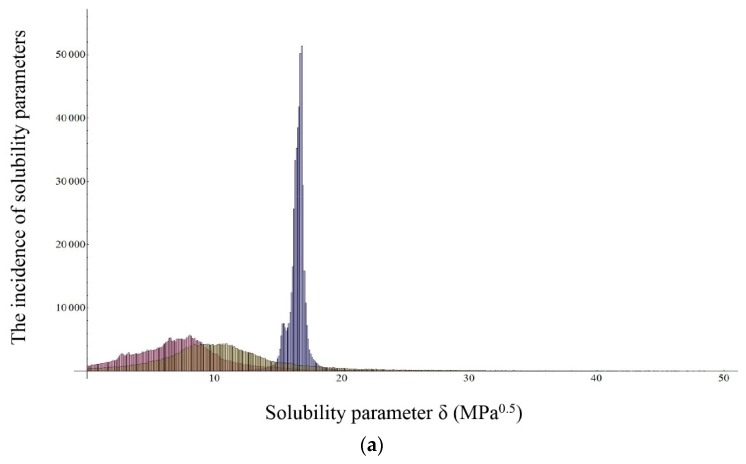

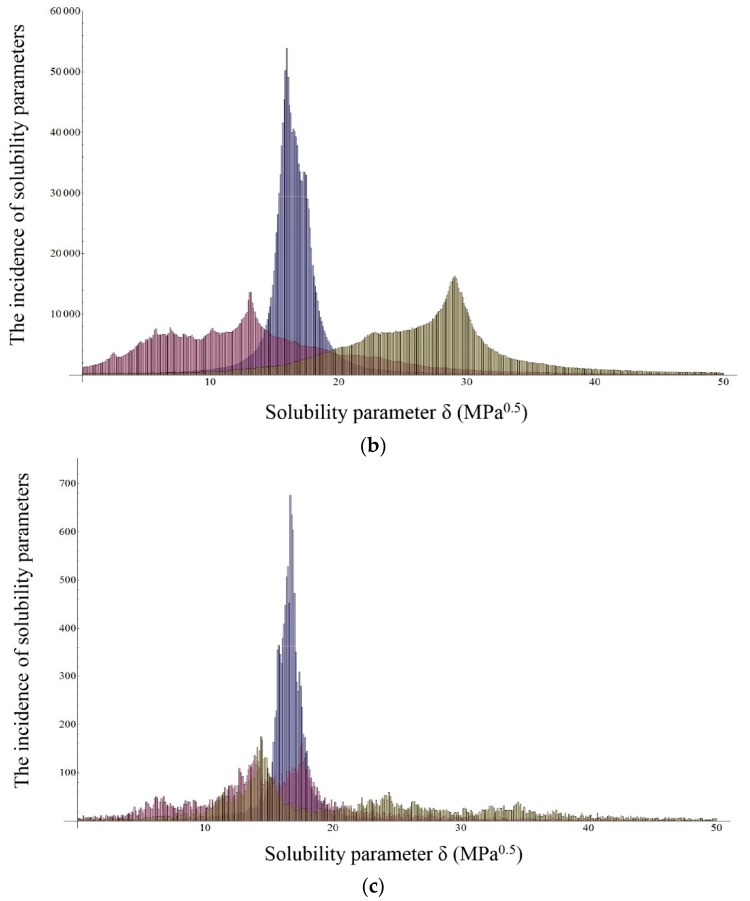
Histograms of most common values of partial solubility parameters obtained by Extended Hansen’s Approach for (**a**) ibuprofen; (**b**) sodium ibuprofenate; (**c**) ibuprofen lysinate; blue color represents values of δ_d_, red color represents values of δ_p_ and green color represents values of δ_h_.

In addition, histograms of all three drugs were drawn by means of the results of regression analysis and are presented in Figure 2. By processing the histograms, we evaluated the most common value for individual solubility parameter and compared it with the calculated median value. Also, histograms were used in comparison of solubility parameters between ibuprofen and it salts, sodium ibuprofen and ibuprofen lysinate. By comparing all three histograms, it can be seen that dispersive solubility parameter takes nearly the same values in all three cases. The values of δ_d_ are highly condensed in a particular area, resulting in greater reliability of estimation of the dispersion solubility parameter. When comparing the histogram of ibuprofen to that of its salts, one can observe an expected shift of values of the polar and hydrogen bonding solubility parameters to higher values. Movements are in line with the increasing hydrophilicity of the ibuprofen molecule after adding lysine and sodium ions.

The choice of solvents can affect the histogram outcome. Due to the smaller number of solvents used in the case of ibuprofen lysinate, the data in the histogram seems to be more scattered in comparison with the histogram of ibuprofen and sodium ibuprofenate. The broader the distribution, the less reliable the reading on the histogram.

#### 2.2.2. Inverse Gas Chromatography (IGC)

Inverse gas chromatography is considered to be one of the most reliable methods for the determination of solubility parameters. There are many published research papers where the solubility parameters of excipients and drugs were successfully determined [29,30,31,32,36]. However, there are none where the method of inverse gas chromatography was used in the determination of solubility parameters of active salts. Therefore, our task was to examine the possibility of using IGC in order to obtain the solubility parameters of an active salt.

**Figure 3:**
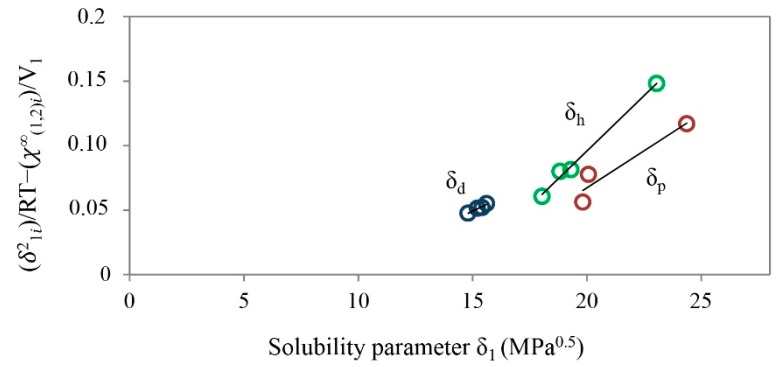
Graph (δ^2^_1i_)/RT − (χ^∞^_(1,2)i_)/V_1_ for ibuprofen lysinate as a function of total solubility parameter of solvents, δ_1_, at T = 303.15 K; the contribution of δ_d_ is marked in blue color, δ_p_ in red color and δ_h_ in green color.

As mentioned in the method introduction, the solubility parameter of given substance can be calculated using Equation (13) by two approaches: as a slope of a straight line or by the intersection of the straight line with the abscissa. Both approaches can only be used in case where linear fitting can be performed for the data points. On that occasion, the contributions of polar and hydrogen bonding solubility parameters are small. It is clear from the very structure of ibuprofen lysinate molecule that this is not going to be the case and one can easily predict a larger contribution to the polar and hydrogen bonding solubility parameter. We have shown this in practice, since the results couldn’t be presented as a single straight line. Due to inability of such presentation, straight lines for each group of solvents were made. Solvents were divided into three groups, as described in the Experimental section, and gave three straight lines, as shown in Figure 3.

The first group of solvents, where *n*–alkanes were classified, represents the contribution of the dispersion solubility parameter and is marked in blue color. The second group of solvents is the group with predominance of the contribution of polar solubility parameter and is marked in red color. The third group, where the contribution of hydrogen bonding solubility parameter is predominant, is marked in green color. Given the fact that obtained straight lines have different slopes, calculation of solubility parameters of ibuprofen lysinate was made using the approach of intersection with the abscissa for each straight line separately. Partial solubility parameters were calculated for all temperatures, at which the IGC was implemented. Results were extrapolated and solubility parameters were determined at T = 25 °C. Results are shown in Table 5.

**Table 5:** Solubility parameters of ibuprofen, taken from [36], and ibuprofen lysinate obtained by using inverse gas chromatography.

Drug	Solubility Parameters			
	δ_d_	δ_p_	δ_h_	δ_t_
Ibuprofen	12.90	6.30	12.30	18.90
Ibuprofen lysinate	15.74	19.18	24.92	35.17

## 3. Discussion of Results

The solubility parameters for ibuprofen and ibuprofen lysinate obtained from all used approaches are summarized in Table 6. For further discussion of solubility parameters, the median value of the group contribution methods for ibuprofen was chosen, as the results don’t differ significantly among methods. In the case of ibuprofen lysinate, both experimental methods were taken into consideration, since one of the main goals was also the comparison between them as tools for the accurate determination of solubility parameters.

**Table 6:** The values of solubility parameters of ibuprofen and ibuprofen lysinate obtained by the calculation approach and by using experimental methods.

Drug	Method		Solubility Parameters (MPa^0.5^)			
			δ_d_	δ_p_	δ_h_	δ_t_
Ibuprofen	*Group contribution method*					
	o Median		17.56	3.22	8.31	19.71
	*Experimental*					
	o Extended Hansen’s approach		16.60	6.91	9.97	20.56
	o Inverse gas chromatography		12.90	6.30	12.30	18.90
Ibuprofen lysinate	*Experimental*					
	o Extended Hansen’s approach	16.97		22.75	12.83	31.15
	o Inverse gas chromatography	15.74		19.18	24.92	35.17

### 3.1. Three-Dimensional Evaluation

For three-dimensional evaluation (3D) of solubility parameters, the approach of Hoftyzer and Van Krevelen was used. The position of substances and distance between them in three-dimensional space were determined using Equation (4). According to the authors, a distance smaller than 5 MPa^0.5^, indicates potential solubility of a given drug in the tested excipient or polymer.

**Figure 4:**
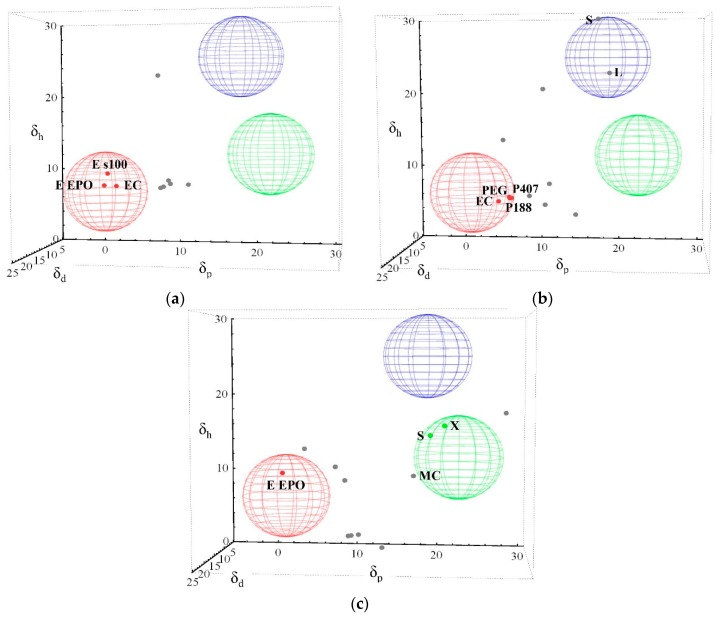
The three-dimensional evaluation of solubility parameters taken from Hoftyzer and Van Krevelen; values of partial solubility parameters for excipients and polymers determined using method authored by (**a**) Hoftyzer and Van Krevelen; (**b**) Stefanis and Panayiotou and (**c**) Just and Breitkreutz; The spheres represent the area of solubility of a given drug, where red represents ibuprofen, green ibuprofen lysinate, whose values of solubility parameters were obtained using EHA and blue ibuprofen lysinate, whose values of solubility parameters were obtained with the IGC method. EC—ethylcellulose; L—lactose; MC—methylcellulose; S—sucrose; X—xylitol; Eudragit E PO, P (188, 407) Poloxamers.

Figure 4 shows 3D spaces, in which drugs, polymers and excipients were placed, according to their partial solubility parameters. Polymers and excipients are marked as grey points, while ibuprofen and ibuprofen lysinate are in the form of spheres with a radius of 5 MPa^0.5^. The sphere represents the area of solubility of a given drug, where red represents ibuprofen, green ibuprofen lysinate, whose values of solubility parameters were obtained using EHA and blue ibuprofen lysinate, whose solubility parameters value were obtained with the IGC method. In the case that the distance between drug and polymer or excipient was lower than 5 MPa^0.5^, the interaction in three-dimensional space is specifically colored.

In the case of ibuprofen, at least one of the tested polymers or excipients is within the range of solubility or in the immediate vicinity, irrespective of the group contribution method used for determination of partial solubility parameters of polymers and excipients (Figure 4). The biggest amount of polymers and excipients within the distance of ≤5 MPa^0.5^ is obtained by using method developed by Stefanis and Panayiotou (Figure 4b). In this case the solubilization of ibuprofen in PEG 6000, Poloxamer 188, Poloxamer 407 and ethylcellulose can be expected. In general, one can observe that different group contribution methods gave slightly different solubility predictions. For example, when using method of Hoftyzer and Van Krevelen, solubility of ibuprofen in Eudragit E PO, Eudragit S 100 and ethylcellulose is estimated (Figure 4a), and when using the method authored by Just and Breitkreutz, the estimation of solubility is limited only to Eudragit E PO (Figure 4c).

As it can be seen in Figure 4, the positions of ibuprofen lysinate spheres are different due to differences in the values of the partial solubility parameters. The difference in estimating the solubility of the drug in the 3D space is expected as well. In the case of ibuprofen lysinate, marked as a blue sphere, none of the excipients or polymers got close enough to be evaluated in regards of solubility. By using method authored by Stefanis and Panayiotou, only lactose and sucrose can be observed in the immediate vicinity to the sphere (Figure 4b). In the case of ibuprofen lysinate, marked as a green sphere, solubility in xylitol and sucrose can be estimated by using the method of Just and Breitkreutz (Figure 4c). Methylcellulose is also located near the ibuprofen lysinate sphere.

### 3.2. Two-Dimensional Evaluation

**Figure 5:**
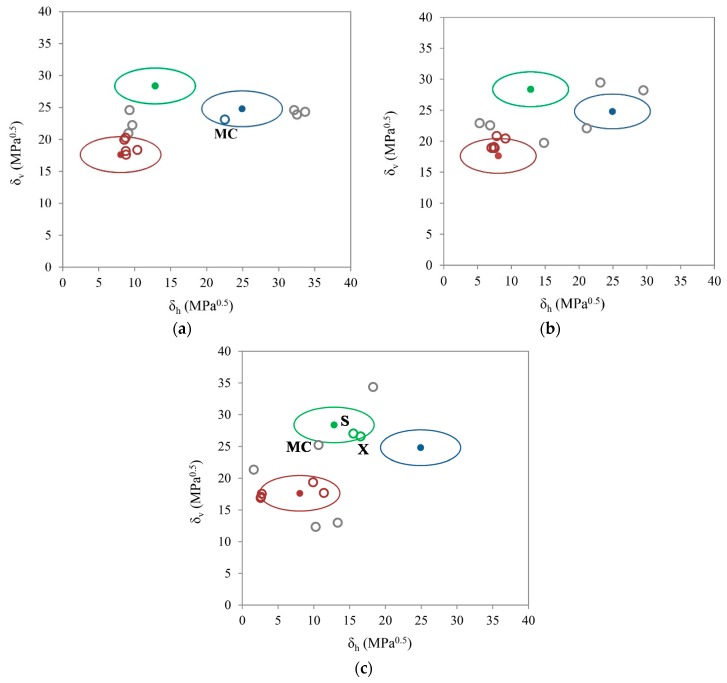
Bagley’s plot; values of solubility parameters of polymers and excipients according to (**a**) Hoftyzer and Van Krevelen; (**b**) Stefanis and Panayiotou and (**c**) Just and Breitkreutz; polymers and excipients are marked as grey circles, while ibuprofen is presented as a red ellipse, ibuprofen lysinate, whose values of solubility parameters were obtained using EHA, is presented as a green ellipse and ibuprofen lysinate, whose values of solubility parameters were obtained by using the IGC method, is presented as a blue ellipse. MC—methylcellulose; S—sucrose; X—xylitol.

The solubility of given drugs in selected excipients and polymers can also be presented on a two-dimensional (2D) level. For such an evaluation, Bagley’s plot was chosen. Given the Equation (6), an ellipse form is obtained in the graphical presentation (Figure 5). The center of the ellipse represents the given drug and the area within a radius of 5.6 MPa^0.5^ represents the range of solubility. Bagley’s diagrams in Figure 5 differ according to the group contribution method choice used in determination of solubility parameters of polymers and excipients. Polymers and excipients are marked as grey circles, while ibuprofen is presented as a red ellipse, ibuprofen lysinate, whose values of solubility parameters were obtained using EHA, is presented as a green ellipse and ibuprofen lysinate, whose values of solubility parameters were obtained by using the IGC method, is presented as a blue ellipse.

In Figure 5 it is clearly shown that a considerable number of polymers and excipients are in the area of the ibuprofen ellipse in all three Bagley plots. This indicates a potentially good solubility of ibuprofen in these materials. All three Bagley plots predict the solubility of ibuprofen in Poloxamer 188 and Poloxamer 407. In addition, two out of three diagrams also estimate its solubility in ethylcellulose, Eudragit S 100 and PEG 6000.

As it can be seen in Figure 5, the positions of the ibuprofen lysinate ellipses are different due to the differences in the values of the solubility parameters. In the case of the blue ellipse, when using the method authored by Hoftyzer and Van Krevelen, methylcellulose appears in the solubility area (Figure 5a). In the immediate vicinity of the blue ellipse, when using method authored by Stefanis and Panayiotou, xylitol also appears. In the case of the green ellipse of ibuprofen lysinate, when using method of Just and Breitkreutz a distance ≤5.6 MPa^0.5^ is calculated only for xylitol, sucrose and methylcellulose (Figure 5c).

### 3.3. One-Dimensional Evaluation

**Figure 6:**
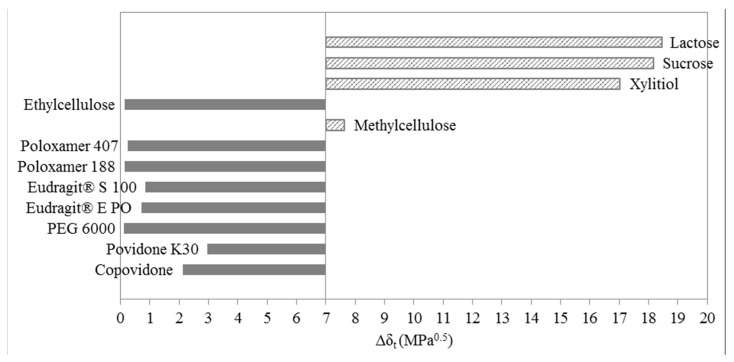
Bar graph for ibuprofen solubility according to Greenhalgh.

The correlation between the solubility parameters of drugs and polymers and excipients can also be presented in one-dimensional (1D) form. For the 1D approach to the solubility, Greenhalgh’s approach was chosen (Equation (7)). The one-dimensional approach only takes into consideration the difference between total solubility parameters. The difference should not exceed 7 MPa^0.5^ in regards of solubility. Figure 6 illustrates a bar graph of the absolute difference between solubility parameters of ibuprofen and selected polymers and excipients. According to Greenhalgh’s approach, solubility of ibuprofen was predicted in the majority of selected substances, with the exception of lactose, sucrose, xylitol and methylcellulose.

**Figure 7:**
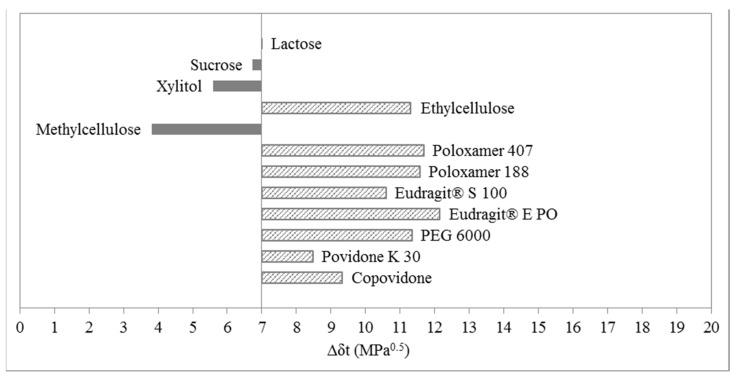
Bar graph according to Greenhalgh for ibuprofen lysinate; solubility parameters of ibuprofen lysinate were obtained using EHA.

**Figure 8:**
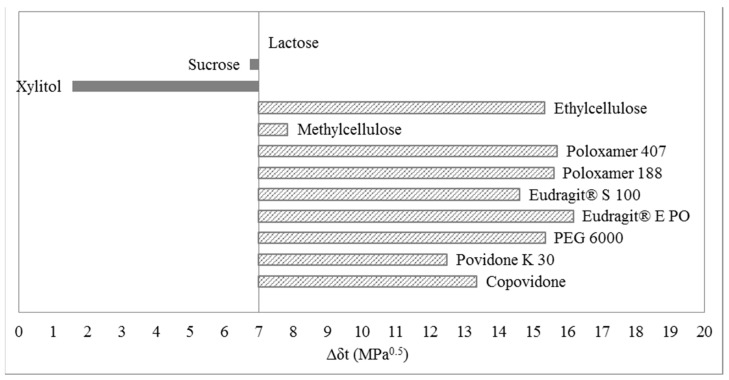
Bar graph according to Greenhalgh for ibuprofen lysinate; solubility parameters of ibuprofen lysinate were obtained using IGC.

Bar graphs of the solubility evaluation of ibuprofen lysinate in selected polymers and excipients are shown in Figure 7 and Figure 8. One can observe a common trend around the upper limit of solubility in both cases, where ibuprofen lysinate is estimated to be soluble in lactose, sucrose, xylitol and methylcellulose. Therefore, one-dimensional solubility prediction, according to Greenhalgh, gives comparable results between the two methods for determination of solubility parameters of ibuprofen lysinate–EHA and IGC. In the case, when EHA was used as a method for determination of solubility parameters (Figure 7), the lowest difference in the total solubility parameter, Δδ_t_, was calculated with methylcellulose. The value is Δδ_t_ = 3.81 MPa^0.5^. In the case of using IGC method for determination of solubility parameters (Figure 8), the lowest difference in the total solubility parameter, Δδ_t_, was calculated with xylitol. The value is Δδ_t_ = 1.56 MPa^0.5^.

## 4. Materials and Methods

### 4.1. Materials

**Figure 9:**
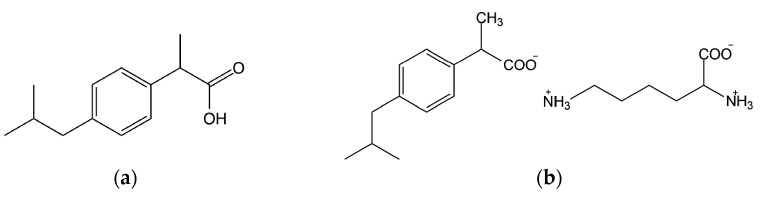
(**a**) Ibuprofen; (**b**) ibuprofen lysinate.

For the purpose of this work, white crystalline powders of ibuprofen and ibuprofen lysinate were used. The chemical structures of the two substances are shown in Figure 9. Substances were supplied by Zentiva, a part of the Sanofi group (Prague, Czech Republic). The following solvents of analytical grade were chosen for implementation of the extended Hansen approach: 1,2-propanediol, 1-butanol, 1-propanol, absolute ethanol, acetic acid, acetone, acetonitrile, dichloromethane, diethyl ether, dioxane, ethyl acetate, ethylene glycol, formamide, heptane, methanol, tetrahydrofuran and distilled water. The following solvents were chosen for implementation of inverse gas chromatography: 1-butanol, acetone, acetonitrile, ethyl acetate, hexane, chloroform, methane, nonane, octane, tetrahydrofuran and toluene.

### 4.2. Methods

#### 4.2.1. Calculation Methods

##### Group Contribution Methods (GCM)

Solubility parameters for the drug ibuprofen and several excipients typically used in hot melt processes, were calculated using six different group contribution methods, authored by: Hansen and Beerbower [37], Fedors [38], Hoftyzer and Van Krevelen [3], Hoy [39], Stefanis and Panayiotou [40], and Just and Breitkreutz [41]. Group contribution methods, authored by Hansen and Beerbower, Fedors and Hoy, gave a value of total solubility parameter, δ_t_, directly, whilst other three group contribution methods, authored by Hoftyzer and Van Krevelen, Stefanis and Panayiotou and Just and Breitkreutz, gave the values of partial solubility parameters. The total solubility parameter, δ_t_, is calculated using Equation (3), if needed. An example of calculation of solubility parameters for a drug ibuprofen and a polymer polyvinylpyrrolidone, using one of the group contribution methods, is shown in Table 7 and Table 8, respectively.

**Table 7:** Calculation of solubility parameters for the drug ibuprofen, using the group contribution method of Hoftyzer and Van Krevelen.

Individual Functional Group	Frequency	F_di_ (MJ/m^3^)^0.5^·mol^−1^	F_pi_^2^ (MJ/m^3^)^0.5^·mol^−1^		E_hi_ (J/mol)	V_m_ (cm^3^/mol)
–CH_3_	3	420 × 3	0		0	33.5 × 3
>CH–	2	80 × 2	0		0	−1.0 × 2
–CH_2_–	1	270	0		0	16.1
phenylene (p)	1	1270	110^2^		0	52.4
–COOH	1	530	420^2^		10,000	28.5
Sum	-	3490	188,500		10,000	195.5
Calculations and results	$\delta_{d}=\frac{\sum F_{di}}{V_{m}}$			δ_d_ = 17.85		
	$\delta_{p}=\frac{\sqrt{\sum F_{pi}^{2}}}{V_{m}}$			δ_p_ = 2.22		
	$\delta_{h}=\sqrt{\frac{\sum E_{hi}}{V_{m}}}$			δ_h_ = 7.15		
	$\delta_{t}^{2}=\delta_{d}^{2}+\delta_{p}^{2}+\delta_{h}^{2}$			δ_t_ = 19.36		

**Table 8:** Calculation of solubility parameters for the polymer polyvinylpyrrolidone, using the group contribution method of Hoftyzer and Van Krevelen.

Individual Functional Group	Frequency	F_di_ (MJ/m^3^)^0.5^·mol^−1^	F_pi_^2^ (MJ/m^3^)^0.5^·mol^−1^		E_hi_ (J/mol)	V_m_ (cm^3^/mol)
–CH_2_–	4	270 × 4	0		0	16.1 × 4
>CH–	1	80	0		0	−1.0
–N<	1	20	800^2^		5000	−9.0
>C=O	1	290	770^2^		2000	10.8
ring (5)	1	190	-		-	16.0
Sum	-	1660	1,232,900		7000	81.2
Calculations and results	$\delta_{d}=\frac{\sum F_{di}}{V_{m}}$			δ_d_ = 20.44		
	$\delta_{p}=\frac{\sqrt{\sum F_{pi}^{2}}}{V_{m}}$			δ_p_ = 13.67		
	$\delta_{h}=\sqrt{\frac{\sum E_{hi}}{V_{m}}}$			δ_h_ = 9.28		
	$\delta_{t}^{2}=\delta_{d}^{2}+\delta_{p}^{2}+\delta_{h}^{2}$			δ_t_ = 26.28		

#### 4.2.2. Experimental Approach

##### Extended Hansen Approach (EHA)

The solubility of ibuprofen lysinate was determined in 17 different solvents, chosen to represent various chemical classes and to cover a wide range of the solubility parameter scale. An excess of the drug was added to the solvent in a conical flask equipped with a ground-glass joint. The stoppered conical flasks were allowed to stir on a magnetic stirplate for 48 h, at T = 25 °C, until the equilibrium solubility had been attained. The suspensions were then filtered through PTFE or RC filters, depending on the solvent used, with pore size of 0.45 μm. 2 mL of saturated solution was pipetted into three different glass flask and rotary evaporated. Distilled water (5–10 mL) was added to the dry residue and absorbance analyzed at 273 nm by using a UV-VIS spectrophotometer (Agilent HP UV-VIS 8453, HP Agilent Technologies Deutschland, Germany). In the case of non-volatile solvents, such as 1,2-propandiol, ethylene glycol, formamide and acetic acid, individual calibration curves were plotted. The densities of saturated solutions were determined using a 10 mL pycnometer, at T = 25 °C, three times for each saturated solution All the data from solubility measurements were used in further calculation of partial solubility parameters in Wolfram Mathematica, by using Equation (8) and a specific regression model. The applied regression model was a linear regression for all possible combinations of eight different solvents among all tested solvents.

##### Inverse Gas Chromatography (IGC)

Inverse gas chromatography was implemented on GC SMS (Agilent Technologies 6890N, Agilent Technologies, Santa Clara, CA, USA), using the following conditions: helium, with a flow rate of 5 mL/min was used as a carrier gas, methane was used as a non-interacting marker in order to correct the dead time retention, the measurements were carried out at four different temperatures, 303.15, 313.15, 323.15 and 333.15 K, with 13 different solvents. The solvents were chosen and classified into three groups, according to the ability to form different intermolecular interactions. Solvents in the first group (*n*-alkanes) were only able to interact with dispersion forces. In the second group were placed solvents with predominant ability to form polar interactions: acetone, acetonitrile and toluene. The third group included solvents which were capable of forming hydrogen bonds like tetrahydrofuran, ethyl acetate, chloroform and 1-butanol. Three injections of the vapor of each solvent were made for each probe at each temperature and the retention time was determined from the maximum of the symmetric peak. By knowing the retention time values of each solvent, we were able to evaluate partial solubility parameters for our sample, ibuprofen lysinate.

## 5. Conclusions

The determination of solubility parameters of a drug in a salt form is still in its beginnings. When dealing with such salts, determination is limited to experimental approaches only, such as the use of the extended Hansen approach (EHA) and inverse gas chromatography (IGC). Within the context of solubility parameter determination, the main focus was on experimental determinations for ibuprofen lysinate and critical evaluation of the results. The values of the total solubility parameter, δ_t_, did not differ significantly among methods, whereas, the values of partial solubility parameters deviated. The largest deviation was estimated in the case of the polar, δ_p_, and hydrogen bonding, δ_h_, solubility parameters. Since the line between both of the aforementioned solubility parameters is rather thin, the better selection and greater number of solvents, used in EHA and IGC, could give us smaller deviations and more reliable data. Due to the simpler nature and structure of the ibuprofen molecule, we were able to use a calculation approach in order to obtain solubility parameter values. The calculation results were in good agreement when compared to experimental data, regarding all three solubility parameters. The interpretation of these results was nevertheless rather challenging, due to the complex behavior of salts in the presence of solvents. The existence of reliable models for salts, on which one could rely, is negligible so far.
